# Robotic Localization Based on Planar Cable Robot and Hall Sensor Array Applied to Magnetic Capsule Endoscope

**DOI:** 10.3390/s20205728

**Published:** 2020-10-09

**Authors:** Min-Cheol Kim, Eui-Sun Kim, Jong-Oh Park, Eunpyo Choi, Chang-Sei Kim

**Affiliations:** 1 School of Mechanical Engineering, Chonnam National University, Gwangju 61186, Korea; kmc100291@gmail.com (M.-C.K.); jop@kimiro.re.kr (J.-O.P.); eunpyochoi@jnu.ac.kr (E.C.); 2 Korea Institute of Medical Microrobotics, Gwangju 61011, Korea; kes@kimiro.re.kr

**Keywords:** augmented sensor with robotic system, Hall effect sensor, cable driven parallel robot, capsule endoscope localization

## Abstract

Recently an active locomotive capsule endoscope (CE) for diagnosis and treatment in the digestive system has been widely studied. However, real-time localization to achieve precise feedback control and record suspicious positioning in the intestine is still challenging owing to the limitation of capsule size, relatively large diagnostic volume, and compatibility of other devices in clinical site. To address this issue, we present a novel robotic localization sensing methodology based on the kinematics of a planar cable driven parallel robot (CDPR) and measurements of the quasistatic magnetic field of a Hall effect sensor (HES) array. The arrangement of HES and the Levenberg-Marquardt (LM) algorithm are applied to estimate the position of the permanent magnet (PM) in the CE, and the planar CDPR is incorporated to follow the PM in the CE. By tracking control of the planar CDPR, the position of PM in any arbitrary position can be obtained through robot forward kinematics with respect to the global coordinates at the bedside. The experimental results show that the root mean square error (RMSE) for the estimated position value of PM was less than 1.13 mm in the X, Y, and Z directions and less than 1.14° in the θ and φ orientation, where the sensing space could be extended to ±70 mm for the given 34 × 34 mm^2^ HES array and the average moving distance in the Z-direction is 40 ± 2.42 mm. The proposed method of the robotic sensing with HES and CDPR may advance the sensing space expansion technology by utilizing the provided single sensor module of limited sensible volume.

## 1. Introduction

Endoscopic visual inspection is a standard diagnostic technique in the clinic for digestive organs. As an advanced technology to reduce pain and risk during endoscopic procedures, a new endoscope concept in the form of a pill equipped with a micro-camera, known as capsule endoscope (CE) was developed [1]. After the CE is swallowed, it moves through the digestive system, allowing doctors a visual diagnostic view inside it. The CE has been improved by the use of new techniques, such as an active locomotion CE that uses the magnetic force between a permanent magnet (PM) inside the CE and an externally controllable magnetic field [2,3,4,5]. Because the CE can be controlled externally, various functional applications, such as tattooing [6,7], biopsy [8], and drug delivery to the affected area [9,10,11] have been presented. 

The main functionality of a CE is to take the digestive organ pictures using an installed camera for visual diagnosis. However, if there is a sudden peristaltic movement in the intestine, including body posture changes during examination [12,13], the CE’s movement also rapidly changes; thus, the CE misses its position that can be matched with the obtained image sets. Therefore, doctors need to obtain accurate location information of the CE in real-time. Moreover, the position of CE is essentially required to achieve precise diagnostics and multi-functionalities control through position feedback of the CE. 

**Figure 1:**
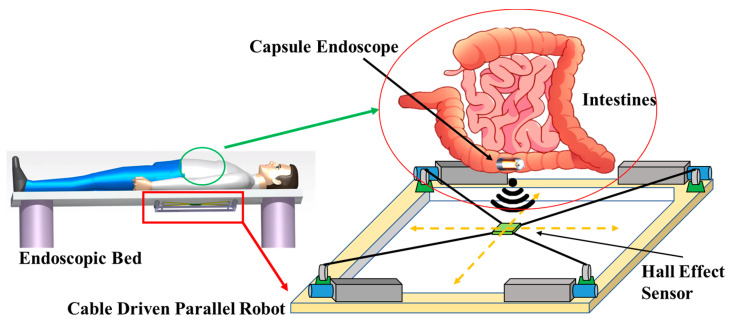
Schematic of the proposed position tracking system capable of sensing volume extension by using CDPR and HES, and its application to CE localization.

Technically, there exist common 3D positioning tracking systems such as vision, ultrasonic, magnetic system, etc. A vision-based system is highly accurate and is capable of tracking certain markers without installing additional systems. However, it is expensive and difficult to detect when certain markers are hidden by obstacles such as inside the human body. The ultrasonic system requires a separate system to transmit ultrasonic waves from the target that sensors can detect [14]. In addition, valid results can only be obtained if the distance between the target and the sensor is above a particular level filled with water or gel. Compared to these device, a magnetic system has less accuracy, however, due to their penetration ability, low cost and configuration simplicity, several types of magnetic sensors are used for 3D positioning of a magnetic object in diverse applications such as to track the position of a CE, or predict soil liquefaction and landslides [15]. For the CE application, MPEG-7 images of the camera installed in the CE and virtual colonoscopy are used to estimate the location of the CE [16] and a CE navigating device is developed and used by the magnetic field control robot system [17,18]. However these methods have cons of necessitating an additional circuit inside the CE or massive equipment to track the position of the CE [19,20] as a large system that typically includes a robot manipulator, external permanent magnet and a vision system is required, which takes up a lot of space. On the contrary our proposed system has the advantage of being installed in a space where CE can be detected from under the patient’s bed, as shown in Figure 1, not only increasing the spatial efficiency but also making it easier to install on an existing bed.

Alternatively, by utilizing the active locomotive ability of a CE containing a PM inside, position recognition methods through magnetic field measurements have been utilized as a magnetic tracking system [21,22,23,24,25]. Based on the PM tracking system, several methods were reported for the CE localization application. Most methods used to estimate the position of CE are based on the principle that the position can be determined by detecting the magnetic field emitted by PM or electromagnets located inside the CE. Simply, if we can theoretically express the strength or the magnetic flux density of the magnet at a random point from the PM, then the relative position of the PM can be estimated by measuring the strength or the magnetic flux density of the magnet at a random point. Thus, many studies have developed algorithms that can estimate the location and direction of PM using relatively inexpensive Hall effect sensors (HESs) to estimate the CE position [26,27,28,29,30,31,32]. However, the previous HES methods require large space for installation and can interfere with other clinical devices such as angiographs, so they need to be ported to a more practical device. 

To overcome these issues in the previous developments, in this paper we propose a novel localization methodology for an actively locomotive CE by utilizing the magnetic properties of a PM inside the CE. Based on the position recognition of the CE that can be obtained through a HES [33,34], the proposed system is designed to utilize kinematics of a planar cable driven parallel robot (CDPR) and measurements of the quasistatic magnetic field by a HES array. The arrangement of HES and the Levenberg-Marquardt (LM) algorithm are applied to estimate the position of the PM in the CE, and the planar CDPR is incorporated to track the position of the PM in the CE. By tracking control of the planar CDPR, the position of PM in an arbitrary position can be obtained through the robot forward kinematics with respect to the global coordinates of a bed. Since the PM can provide a magnetic field externally and internally through the electromagnetic guide system to actuate the CE [35,36], an electromagnetic actuator (EMA) system consisting of Helmholtz and Maxwell coils or external magnets is also compatible with the proposed localization methodology [37,38]. 

An overview of the proposed method is presented in Figure 1. While examining digestive organs with the CE containing the PM, the CDPR installed under the patient’s bed is operated simultaneously to track the PM. The HES is attached to the end-effector (EE) of the CDPR. When the CE enters the patient’s digestive tract and starts moving, the HES attached to the CDPR can recognize the magnetic field intensity of the PM in the CE and can compute the location of the CE in real time and the position of the CE with respect to the global coordinates of the CDPR can be calculated using the cable length and forward kinematics of the CDPR. Contrary to a conventional CE that has the problem of missing the location of CE owing to the active movement of digestive organs, the proposed methodology can overcome the problem of diagnosis blind spots and may open extensive applications to a wider range of organs. This has the advantage of being able to find patients’ lesions more easily and delivering the exact location of the CE to the doctor, thus, shortening the diagnosis and treatment time.

This paper is organized as follows: Section 2 describes the algorithm and formula for estimating the PM position by HES, and provides a brief introduction about the CDPR’s description, structure, and kinematics. The experimental setup and results are provided in Section 3. Finally, we present conclusions about the experiments for estimating the position of PM by HES with future works of position estimation and tracking system of CDPR.

## 2. PM Position Recognition Method

### 2.1. PM Sensing by Using the HES Array

**Figure 2:**
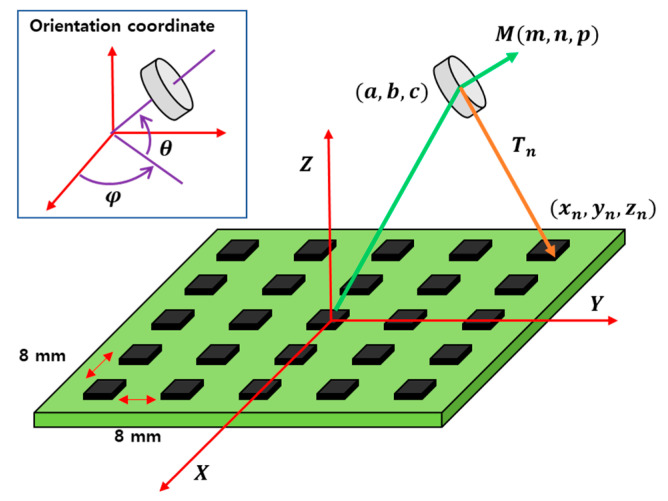
Schematic of HES array and PM; HES array is configured to detect a PM in 3D space.

Through the HES measurement and numerical model, the position of PM can be determined with respect to the center position of the HES array. Thus, a tracking system with the PM placed in the center of the HES array is developed. In addition, the angular posture and the height of the PM are measured by the HES array as shown in Figure 2. 

The HES is a device that measures the magnitude of a magnetic field. A schematic diagram for recognizing the position and directional values of PM located in 3D is depicted in Figure 2. The HES is placed in rows and columns at a certain interval of 8 mm. The total HES array dimension is 34 × 34 mm^2^. It can measure the magnetic field intensity of the PM using a 25 HES array. We define the unit axis coordinate system for X, Y, Z as (i,j,k) and the center orientation and rotation of the PM as (a,b,c,m,n,p). If there are *N* HES, the position of the *i*th HES is $\left(x_{i},y_{i},z_{i}\;\;i=1,2,\ldots ,N\right)$. In Equation (1), $T_{l}$ is the distance from the vector of PM (a,b,c) to each HES $\left(x_{i},y_{i},z_{i}\right)$. At this point, the magnetic field intensity, $B_{l}$, value at the position of the *i*th HES $\left(x_{i},y_{i},z_{i}\right)$ can be obtained as in Equation (5) using Biot–Savart law that is commonly used in magnetic field models for estimating magnetic fields as a magnetic dipole:

(1) $$\vec{T_{i}}=\left(x_{i}-a,y_{i}-b,z_{i}-c\right)$$

(2) $$T_{l}=\sqrt{{\left(x_{i}-a\right)}^{2}+{\left(y_{i}-b\right)}^{2}+{\left(z_{i}-c\right)}^{2}}\;\;\mathrm{for}\;i=1,2,\ldots ,N$$

(3) $$B_{l}=B_{x}i+B_{y}j+B_{z}k$$

where, the rotation value of the PM is defined by $\vec{M}$. The values (m,n,p) are $m^{2}+n^{2}+p^{2}=1$ and we use in Equation (4) to convert $\vec{M}=\left(m,n,p\right)$ values.

(4) m=sin(θ)cos(φ),    n=sin(θ)sin(φ),    p=cos(θ)

The $B_{T}$ is a constant coefficient associated with the magnetic field moment of the magnet, where $\mu_{r}$ is the relative permeability of the medium, $\mu_{0}$ is the air magnetic permeability, $M_{T}$ is the magnetization:

(5) $$B_{T}=\frac{\mu_{r}\mu_{0}M_{T}}{4\pi },\;\;\;\vec{B_{l}}=B_{T}\left(\frac{3\left(\vec{M}\cdot \vec{T_{l}}\right)\vec{T_{l}}}{T_{l}^{5}}-\frac{\vec{M}}{T_{l}^{3}}\right)$$

Finally, the magnetic flux density of the three orthogonal components can be defined as follows:

(6) $$B_{l}=\{\begin{array}{c} B_{xi}=B_{T}\left(\frac{3\left[m\left(x_{i}-a\right)+n\left(y_{i}-b\right)+p\left(z_{i}-c\right)\right]\cdot \left(x_{i}-a\right)}{T_{l}^{5}}-\frac{m}{T_{l}^{3}}\right)\\ B_{yi}=B_{T}\left(\frac{3\left[m\left(x_{i}-a\right)+n\left(y_{i}-b\right)+p\left(z_{i}-c\right)\right]\cdot \left(y_{i}-b\right)}{T_{l}^{5}}-\frac{n}{T_{l}^{3}}\right)\\ B_{zi}=B_{T}\left(\frac{3\left[m\left(x_{i}-a\right)+n\left(y_{i}-b\right)+p\left(z_{i}-c\right)\right]\cdot \left(z_{i}-c\right)}{T_{l}^{5}}-\frac{p}{T_{l}^{3}}\right) \end{array}$$

The magnetic field model for a cylindrical PM is a nonlinear equation with five degrees of freedom, indicating the position and direction of the PM and it is symmetrical to the direction of the length of the PM. Therefore, it is necessary to obtain a nonlinear minimum magnetic flux to estimate the position of the PM. We can obtain a 3N expression when using N three-axis HES. To address, five parameters (x,y,z,θ,φ) representing the position and direction of the magnet need to be obtained by applying a nonlinear least square method to minimize errors in these magnetic flux expressions. For that, the object function is defined as Equation (7), where $B_{iz}^{*}$ is the measured data and $B_{iz}$ is calculated from Equation (6):

(7) $$\begin{array}{c} \arg\min f=\overset{n}{\underset{i=1}{\sum }}\left(\left[\begin{array}{c} B_{xi}\\ B_{yi}\\ B_{zi} \end{array}\right]-B_{T}\left\{\frac{3\left(\vec{M}\cdot \vec{T_{l}}\right)\cdot \vec{T_{l}}}{T_{l}^{5}}-\frac{\vec{M}}{T_{l}^{3}}\right\}\right)\\ \mathrm{and}\\ E_{z}=\overset{25}{\underset{i=1}{\sum }}{\left[B_{iz}^{*}-B_{iz}\right]}^{2} \end{array}$$

To solve the equation of the position and direction of the PM through the magnetic flux density value obtained from HES, [39] compared various nonlinear minimization algorithms about the magnetic flux density and proved that the Levenberg–Marquardt (LM) algorithm is ideal for accuracy and processing speed. The LM algorithm is one of the typical methods used to solve nonlinear least squares problems. However, an initial parameter estimate is required to find the optimal value. Therefore, in this study, the continuous motion of the PM is estimated using the LM algorithm, and assuming that the initial position is correct, estimate calculation speed and accuracy were improved by applying the previous position value to the initial position value, where the initial position is selected that the PM is currently located at the center point of the EE of the CDPR through experiments. To obtain the value of the PM (a,b,c,m,n,p), a Jacobian function is proposed, as shown in Equation (8). The analytical Jacobian derivation for magnetic localization for HES can be found in [40]. The *h* parameter increment is obtained by Equation (9) using the object function $E_{z}$. This calculates the $B_{iz}$ value of the PM obtained and continues to estimate the changing position of PM:

(8) $$J=\left[\begin{array}{cccccc} \frac{\partial B_{1}}{\partial a} & \frac{\partial B_{1}}{\partial b} & \frac{\partial B_{1}}{\partial c} & \frac{\partial B_{1}}{\partial m} & \frac{\partial B_{1}}{\partial n} & \frac{\partial B_{1}}{\partial p}\\ \frac{\partial B_{2}}{\partial a} & \frac{\partial B_{2}}{\partial b} & \frac{\partial B_{2}}{\partial c} & \frac{\partial B_{2}}{\partial m} & \frac{\partial B_{2}}{\partial n} & \frac{\partial B_{2}}{\partial p}\\ \vdots & \vdots & \vdots & \vdots & \vdots & \vdots \\ \frac{\partial B_{25}}{\partial a} & \frac{\partial B_{25}}{\partial b} & \frac{\partial B_{25}}{\partial c} & \frac{\partial B_{25}}{\partial m} & \frac{\partial B_{25}}{\partial n} & \frac{\partial B_{25}}{\partial p} \end{array}\right]$$

(9) $$\left[J\left(y_{k}\right)J^{T}\left(y_{k}\right)+\mu I\right]h=J^{T}\left(y_{k}\right)E_{z}\left(y_{k}\right)$$

where $y_{k}=\left[a_{est},b_{est},c_{est},m_{est},n_{est},p_{est}\right]$ and $y_{k+1}=y_{k}+h$ are used to find the numerical solution. 

### 2.2. PM Tracking by the CDPR

**Figure 3:**
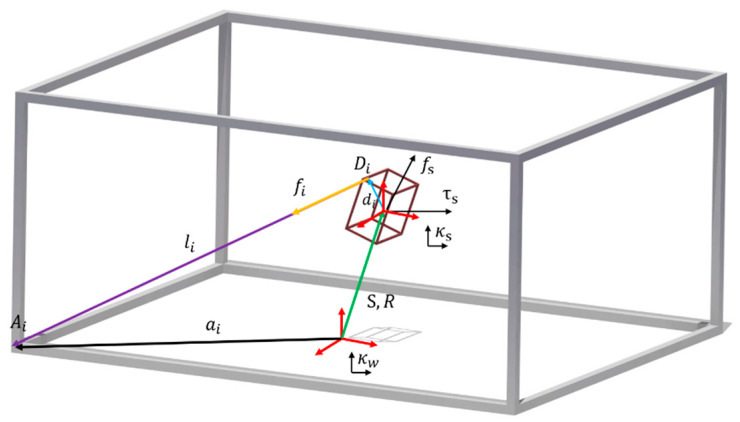
Kinematic diagram of the CDPR.

The CDPR is a special type of parallel robot that moves by elastic cables, not by the robust rigid links utilized for general robot systems as shown in Figure 3. Currently, the most commonly used application is the Sky-cam; however, research on other applications, such as logistics and entertainment CDPRs, is being widely conducted [41,42]. The CDPR consists of a fixed frame, winch-motor, EE, elastic polymer cable, and pulley. Therefore, it is easy to configure a low-cost robot system. Furthermore, by changing the cable length, and the position of the winch, the workspace of the EE can be increased at a minimum cost. Further details can be obtained in [43], where the mechanism and principal details of CDPR in the view of robotics are explained.

We designed the planar CDPR workspace by considering the general patient bed size for endoscopic examination that is approximately $1800\;\left(L\right)\times 700\;\left(W\right)\times 650\;\left(H\right)\;{\mathrm{mm}}^{3}$ and the average abdominal area of typical person that is $534\;\left(L\right)\times 250\;\left(W\right)\times 180\;\left(H\right)\;{\mathrm{mm}}^{3}$ as reported in [44]. Because of the unique characteristics of the CDPR’s wide workspace, the workspace can be expanded to the required size mentioned above. Finally, the planer CDPR is designed to satisfy the workspace based on the average person’s abdominal area and the patient’s bed area as 800 (L)×800 (W) in plane. 

In the previous systems [26], workspace was $70\;\left(L\right)\times 70\;\left(W\right)\times 50\;\left(H\right)\;{\mathrm{mm}}^{3}$ and they tested MACE sensing volume ranges from (20,−20,−35) mm to (−18,18,−20) mm. In contrast, the tracking system proposed in this paper is basically a CDPR system, which can increase the workspace more easily just by changing the frame size and cable length.

In this study, through the tracking performance of CDPR, the position of CE, which is the same with the EE position of CDPR, can be obtained by forward kinematics with respect to the global coordinate of the sensing system. Figure 3 shows the kinematic diagram of the CDPR, where the geometric parameter $A_{i}$ is a proximal attachment point to the winch and $D_{i}$ is the distal attachment point of EE. $K_{w}$ is the world coordinate and local coordinated of the frame and $K_{l}$ is the closure vector loop. $l_{i}$ is a vector representing the cable length of the connection between $A_{i}$ and $D_{i}$. If EE is rotated at angle α,β,γ, we calculate rotation vector R using Z-Y-X Euler angle law. In Equation (12) we calculate the vector of cable length, and then the length of cables is obtained through the Euclidean norm in Equation (13). In Equation (11), s and c represent sine and cosine, respectively. In this study, two types of CDPR were used for the experiment: 3D (X, Y, Z) CDPR and 2D (X, Y) CDPR. The former one is for the CE motion for validation and the latter one is for localization:

(10) $$l_{i}={\left[l_{xi},l_{yi},l_{zi}\right]}^{T}\;\;\;\;\;\mathrm{for}\;i=1,2,\ldots ,N$$

(11) $$R=\left[\begin{array}{ccc} c\beta c\gamma & c\gamma s\alpha s\beta -c\alpha s\gamma & c\alpha c\gamma s\beta +s\alpha s\gamma \\ c\beta s\gamma & c\alpha c\gamma +s\alpha s\beta s\gamma & -c\gamma s\alpha +c\alpha s\beta s\gamma \\ -s\beta & c\beta s\alpha & c\alpha c\beta \end{array}\right]$$

(12) $$l_{i}=A_{i}-S-RD_{i}$$

(13) $$l_{i}=\sqrt{{\left[A_{i}-S-RD_{i}\right]}^{T}\cdot \left[A_{i}-S-RD_{i}\right]}$$

Finally, the forward kinematics for determining the position of CE can be derived as follows: In the X-Y plane, X and Y positions of CE are obtained through forward kinematics based on the cable length values obtained by inverse kinematics, without considering the rotation. Equation (13) can be expressed as shown in Equation (14). Four cables were used for constructing the X-Y plane. Thus, $a_{i}$ and $b_{i}$ are the position vector of CDPR, which are 2 × 4 matrix. In Equation (14), $A_{xi}-D_{xi}$ and $A_{yi}-D_{yi}$ are constant values, and they can be represented as Equation (15). Through four cable length $\left(l_{1},l_{2},l_{3},l_{4}\right)$ simultaneous equation, the position values on the X-Y planes of CE can be expressed as Equation (16). The rotation value of CE is estimated by the spherical coordinate measured at HES in Section 2.1:

(14) $$l_{i}=\sqrt{{\left[A_{xi}-S_{x}-D_{xi}\right]}^{2}+{\left[A_{yi}-S_{y}-D_{yi}\right]}^{2}}$$

(15) $$A_{xi}-D_{xi}=C_{xi},\;\;\;\;A_{yi}-D_{yi}=C_{yi}$$

(16) $$\left[\begin{array}{c} S_{x}\\ S_{y} \end{array}\right]=\frac{1}{2}{\left[\begin{array}{cc} C_{x2}-C_{x1} & C_{y2}-C_{y1}\\ C_{x4}-C_{x3} & C_{y4}-C_{y3} \end{array}\right]}^{T}\left[\begin{array}{c} L_{1}^{2}-L_{2}^{2}-C_{x1}^{2}-C_{y1}^{2}+C_{x2}^{2}+C_{y2}^{2}\\ L_{3}^{2}-L_{4}^{2}-C_{x3}^{2}-C_{y3}^{2}+C_{x4}^{2}+C_{y4}^{2} \end{array}\right]$$

Equation (17) allows a CDPR to track PM’s position where we assume that the X and Y values of the estimated PM in p come from Equation (12). The difference between the X and Y values estimated before and after the PM moves is defined as the tracking position of Equation (17). In Equation (17), previous magnet position, $\hat{P}\left(k-1\right)$, and current magnet position, $\hat{P}\left(k\right)$, represent the value obtained through the pose estimator and forward kinematics, respectively. The tracking position is inserted, as shown in Equation (18), and the calculated cable length are used to track the EE of CDPR to the position where the PM is moving. Because the rotation angle of the cable robot is not provided here, the value of θ is zero, and R is a unit matrix:

(17) $$TP=\hat{P}\left(k-1\right)-\hat{P}\left(k\right)$$

(18) $$l_{i}=A_{i}-\left[\begin{array}{cc} TP & X_{est}\\ TP & Y_{est} \end{array}\right]-RD_{i}$$

(19) $$R=\left[\begin{array}{cc} \cos\left(\theta \right) & -\sin\left(\theta \right)\\ \sin\left(\theta \right) & \cos\left(\theta \right) \end{array}\right]$$

### 2.3. PM Position Estimation

**Figure 4:**
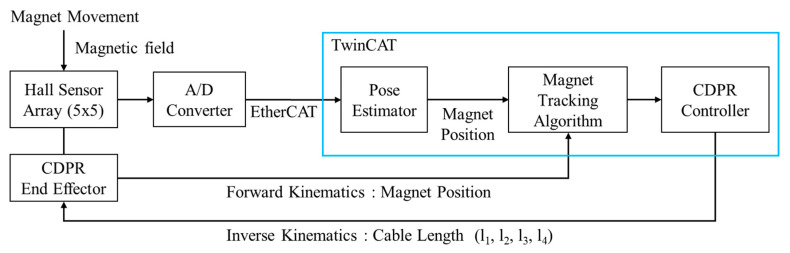
Block diagram for the proposed PM position tracking system.

The PM position is estimated by HES measurements and CDPR kinematics. The block diagram of the whole localization system is shown in Figure 4. 

The procedure is designed as follows: First, an arbitrary position value for the PM to measure the magnetic field at the HES array is created. Second, the A/D Converter is used to transmit the HES value to TwinCAT via EtherCAT. Third, Equations (7)–(9) are used to estimate the pose of the PM. Fourth, we input the estimated position of the PM to the magnet tracking algorithm and then input the PM’s tracking position (17) to the CDPR controller. The CDPR EE is moved by calculating the cable length through inverse kinematics in Equation (18). Finally, we feedback the calculated forward kinematics in Equation (16) to the magnet tracking algorithm, and then the CDPR EE tracks and estimates the position of PM. 

## 3. Experimental Results

### 3.1. Experimental Setup 

**Figure 5:**
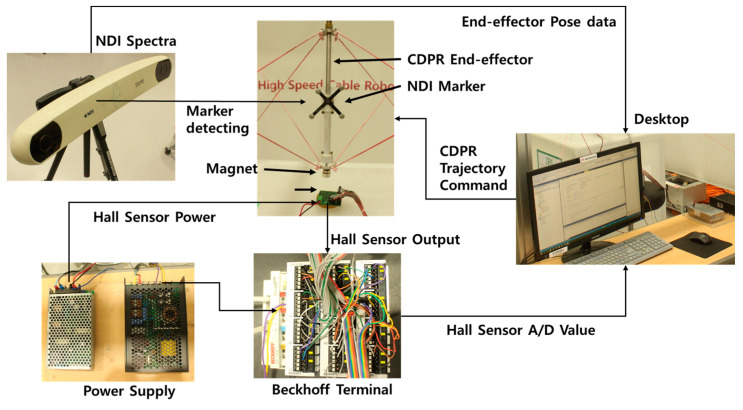
PM position estimation system for HES performance validation experiment.

The experimental setup for the validation of the PM position estimation is shown in Figure 5. 

The system configuration is described in detail below. It consists of a cylindrical PM, a power supply, a control PC, an EtherCAT I/O converter, an NDI Spectra (accuracy 0.25~0.35 mm, maximum update rate 60 Hz, maximum data rate 1.2 Mb/s), a CDPR, and the prototyped 5 × 5 HES array board. To implement the interface of the PM’s position estimation, a Windows PC is utilized for a controlled environment with a cycle time of 10 ms using the TwinCAT 3.1 PLC from the Beckhoff Company (Verl, Germany) where the EtherCAT fieldbus protocol is incorporated. The TwinCAT 3.1 PLC provides IEC 61131-3 language and uses ST language to program CDPR and HES position estimation algorithms. The magnetic flux data of PM is obtained from HES via EtherCAT communication using Beckhoff’s ELM3148, which is operated by receiving 24 V with 25 HES data. The position of the magnet is estimated through the LM algorithm in the TwinCAT 3.1. The PM is cylindrical, with 20 mm diameter and 10 mm height. The PM was rated N45 and the size of the PM is selected by commercially available ones by considering the capsule endoscope size as 11 mm diameter and 26 mm height [3].

The size of the PM can be reduced depending on the HES sensor performance. The HES was a WSH202 from Winson (Hsin-chu, Taiwan) with a linear HES IC. It is operated by receiving 12 V. The HES was 25 with an array of 5 × 5 matrix in the X-Y plane, each with and interval of 8 mm, total dimensions of the HES array is 34×34 mm^2^. The interval between HES was determined based on the available EE size of the planer CDPR (37×37 mm^2^). The number of HES was set in this study at 25 in consideration of the individual size of the HES (2.9 × 1.6 mm^2^). However, the arrangement of the HES will further need to be optimized that necessitates minimum number of HES. However, the size of EE and number of HES arrays can be changed with respect to the clinical requirements. And the CDPR frame is made of plastic and polymer cable is utilized in here, there is no magnetization interference while measuring HES signals. 

The X, Y, and Z values were observed using NDI Spectra as a standard to verify that the EE of the CDPR was moving correctly. NDI markers were installed on the CDPR EE and received values when the CDPR EE moved.

### 3.2. HES Performance Validation 

**Figure 6:**
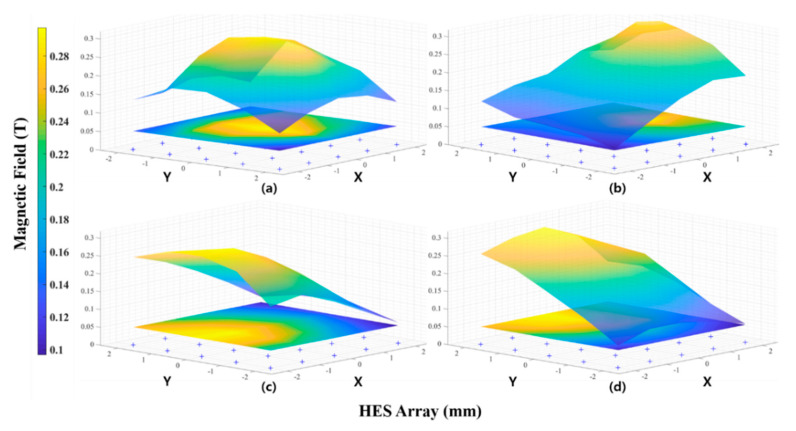
Measured magnetic field of PM using HES with respect to PM’s different positions; (**a**) PM stayed in HES center Point. (**b**) PM stayed in HES +X direction. (**c**) PM stayed in HES -X direction. (**d**) PM stayed in HES +Y direction.

Figure 6 shows the measured magnetic field obtained by moving the PM on an X-Y plane 40 mm above the Z-axis of the HES array. We can see that the equipped HES can detect the position of the magnet on the designed HES array placed in different position.

**Figure 7:**
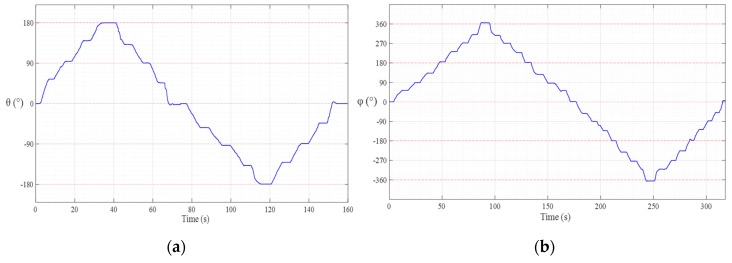
Measured rotation angle of PM using HES with respect to PM’s different rotation; (**a**) PM rotated in θ direction, (**b**) PM rotated in φ direction.

Figure 7 shows the measured rotation angle by changing the PM angle in every 45° on *θ*-*φ* direction (shown in Figure 2) at 40 mm above the HES array. Where we can see that the equipped HES can detect the rotation angle (left: *θ* is changing ±180°, right: *φ* is changing ±360°) of the cylindrical PM on the designed HES array placed in different rotation. 

We utilized six degrees of freedom CDPR in Figure 5 to move the PM in space to provide an accurate reference position for validation measurements from the HES using the LM algorithm. We attached the PM to CDPR EE and positioned the HES to the Z axis, 48 mm below the PM, and the EE was moved to helical (X-Y-Z plane) and rotation (θ, φ) motion. Simultaneously, the accurate position of CDPR EE should be verified. In addition, when estimating the position of PM in each motion, NDI Spectra was used to measure the position of the NDI marker attached to the CDPR EE. 

First, to verify the accuracy of the position estimates of the X, Y, and Z of PM, we moved the CDPR EE with a helical motion. The NDI measured the EE position, the position value of the PM estimated by the HES, and the input value in the trajectory are displayed graphically. 

**Figure 8:**
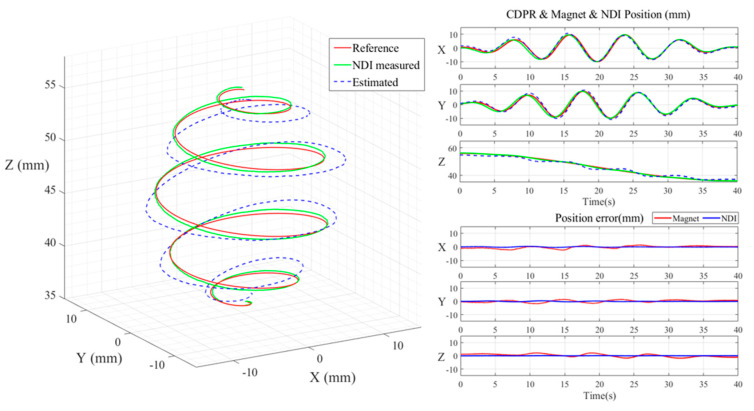
CDPR EE moves to Helical trajectory in the X-Y-Z direction and using NDI Spectra & HES measure CDPR EE’s and PM’s X, Y Z Position (**Left**). NDI Spectra & HES validation in time domain position and error value in each direction (**Right**).

**Figure 9:**
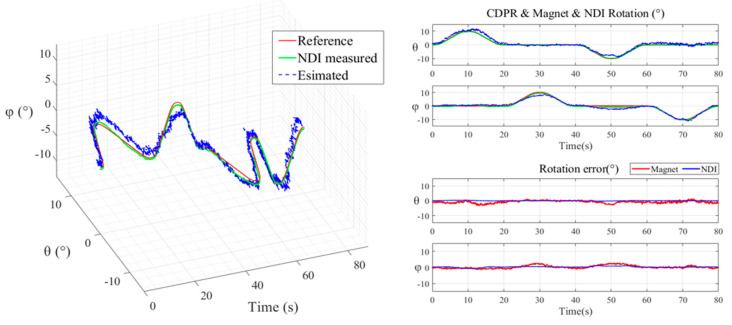
CDPR EE moves to rotation trajectory in the θ-φ direction and using NDI Spectra & HES measure CDPR EE’s and PM’s θ, φ degree (**Left**). NDI Spectra & HES validation in time domain rotation and error value in each direction (**Right**).

First, the helical motion started from point (0,0,50) and moved to point (0,0,35) using a spiral trajectory as shown in Figure 8 that rotated five times for 40 s along a circle with a maximum diameter of 20 mm. Second, the rotational motion used a rotation trajectory that moved (θ, φ) at the center point for 80 s in the ±10° direction as shown in Figure 9.

Table 1 shows the values obtained by root mean square error (RMSE) from the error of the NDI and HES values for X, Y and Z measured by the experiment and the error of the trajectory reference values. The result showed that when the PM was driven in a helical motion, the RMSE values of NDI were 0.1797, 0.1739 and 0.0441 mm at X, Y and Z, respectively, and the RMSE values of HES were 0.9531, 0.8496 and 1.3493 mm at X, Y, and Z, respectively. When the PM was driven in a rotational motion, the RMSE values of NDI were 0.1232° and 0.4875° at θ and φ, respectively, and the RMSE values of HES measurements were 1.1449° and 1.0408° at θ and φ, respectively.

It is expected that the location of the CE which moves in the human body’s digestive organs will be easily identified. Even if the CE moves rapidly owing to some sudden active movement of the intestine, it is believed that the CE’s location can be easily tracked, because the HES can estimate the final missing location of the CE and redetermine the current location of the CE. Therefore, in Section 3.3, we conducted an experiment in which CDPR EE tracks the PM. 

### 3.3. Position Estimation by CDPR Tracking Control 

**Figure 10:**
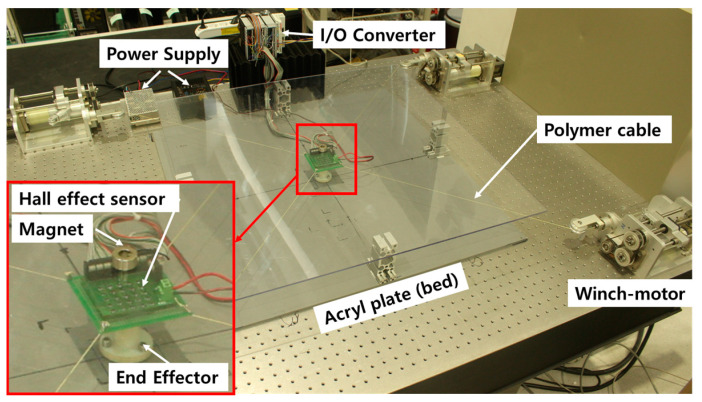
The prototyped system with CDPR EE equipping HES which can track the position of the PM.

The proposed sensor system is rigidly attaching the HES to the center of the EE of the planer CDPR, as shown in Figure 10, to track the CE correctly, after careful alignment to the center of the EE. This experiment setup is almost same as shown in Figure 5, just the CDPR is changed from 3D to 2D and we don’t use the NDI sensor in this experiment. The acrylic plate on the Z axis was installed +40 mm above the HES. The PM was moved randomly on the acrylic plate to enable the CDPR EE to track the PM’s position, that is, the estimated position of the HES. The purpose of this experiment is to show that the CDPR can track the estimated position of PM by HES and CDPR forward kinematics.

To track a randomly moving PM, the CDPR EE with the HES was moved to track the position of the PM. The difference between the previously estimated position of PM, which was obtained through the pose estimator, and its current position, which is obtained through the forward kinematics, is more than 3 mm. The CDPR is moved to track the PM. This tracking range was set at 3 mm based on that the CDPR received data from the HES in real time when the tracking range was set to zero. Thus, even though Low pass filtering was done, the noise data of HES caused high vibration of CDPR EE. Therefore, we used a threshold of the CDPR tracking motion to reduce the mechanical vibration caused by noise signals. 

#### 3.3.1. Tracking Validated for Static Position in Z-axis

**Figure 11:**
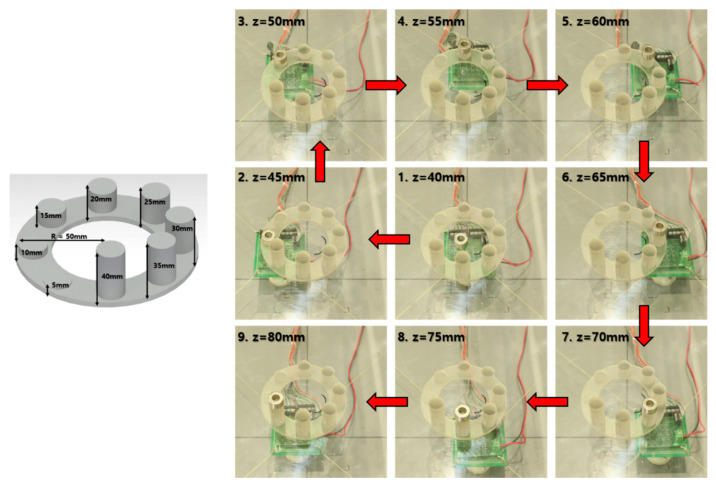
Stereoscopic objects of CDPR tracking system experiment (**Left**) and snapshot of the CDPR tracking system in stereoscopic object jig (**Right**).

The performance of the PM tracking system driven by the CDPR is validated by a stereoscopic jig, as shown in Figure 11, in which eight columns were made at intervals of 45° to from 5 to 40 mm in height. 

The diameter of each cylinder was set at 20 mm for placing a PM, and it was made with a 3D printer. The reason for setting the criteria up to 40 mm is that the limit estimated Z position of the PM was up to 80 mm. Because the current PM is 40 mm away from the HES to the +Z axis, the maximum height of the cylinder is set to 40 mm. In this experiment, the only direction to X and Y is moved when the CDPR tracks the estimated position of PM. To test the exact data on the Z-axis, we created tracking experimental conditions as follows. Figure 11 shows the snapshot of the CDPR tracking the position of the PM while moving the PM sequentially on a column of different heights. 

**Figure 12:**
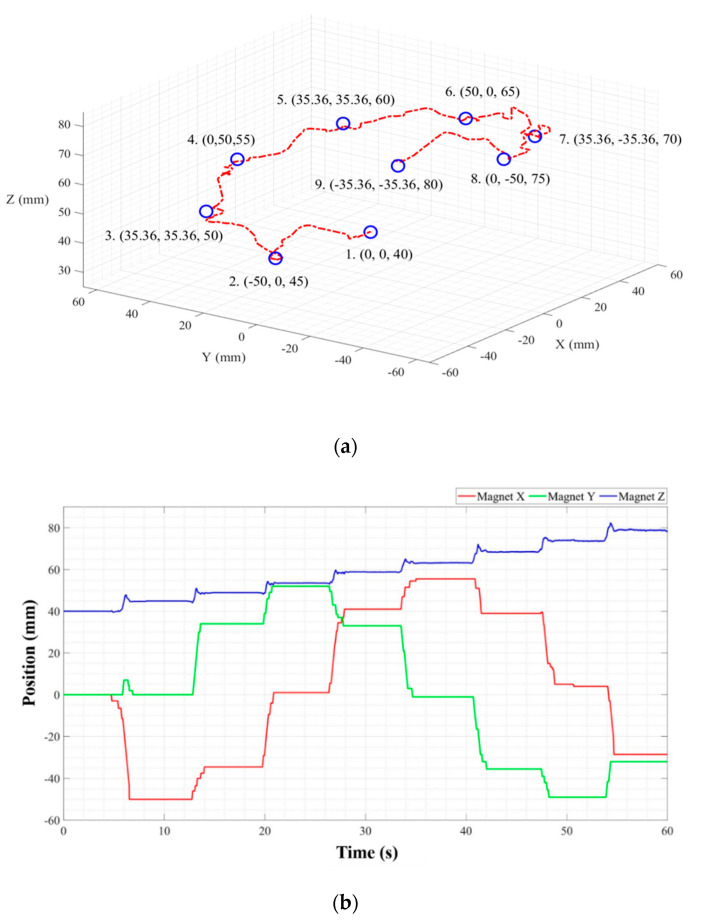
The result of CDPR EE tracking the 3D-object position of PM; (**a**) Tracking and localization performance in 3D space and (**b**) Tracking and localization performance in time domain.

Figure 12a shows the result of a 3D posture of the PM controlled to track the object, where the red line is the path through which PM moved, and the blue circle is the reference coordinates point of the cylinder. Figure 12b is a graph of the X, Y, and Z position of the PM measured at CDPR within a time span; the values X, Y, and Z are the same as the positions in the center of the PM. Simply, the CDPR EE is a tracking system that follows the center point of the PM in the same 2D plane. The estimated Z value of the PM is not tracked by CDPR but only indicates the height. 

To confirm the strength of the proposed method of wide workspace extensibility with high SNR, we quantitatively analyzed the signal to noise ratio (SNR) from the experimental data of static HES (when stopping) and dynamic HES condition (when moving). Where, the PM was moved in Z-axis from 40 mm to 80 mm. The SNR results are presented in Table 2. The range of changes in SNR differed by 49.22 dB at static condition and by 24.09 dB when HES was moving condition. Therefore, we could validate that the SNR of the proposed system has significantly is improved owing to the tracking mechanism.

#### 3.3.2. Tracking Performance Validation

By considering the human intestine model, we utilized the illustration of the human small and large intestines image in this experiment. We printed out 270 (L)×220 (W) mm^2^ in actual size to verify the tracking system while moving the PM on it. Unlike the previous experiments in Section 3.3.1 in which the tracking system was verified according to the static position change in the Z-axis height, the gap between HES and PM was fixed at 40 mm. For the given randomly assigned 1−23 point sets on the actual size intestine image, the PM is moved to follow each point in order and the CDPR was tacking the PM. The 23 points were set based on the home position of the planer CDPR coordinate and the reference trajectory during tracking was obtained through forward kinematics of the planer CDPR.

**Figure 13:**
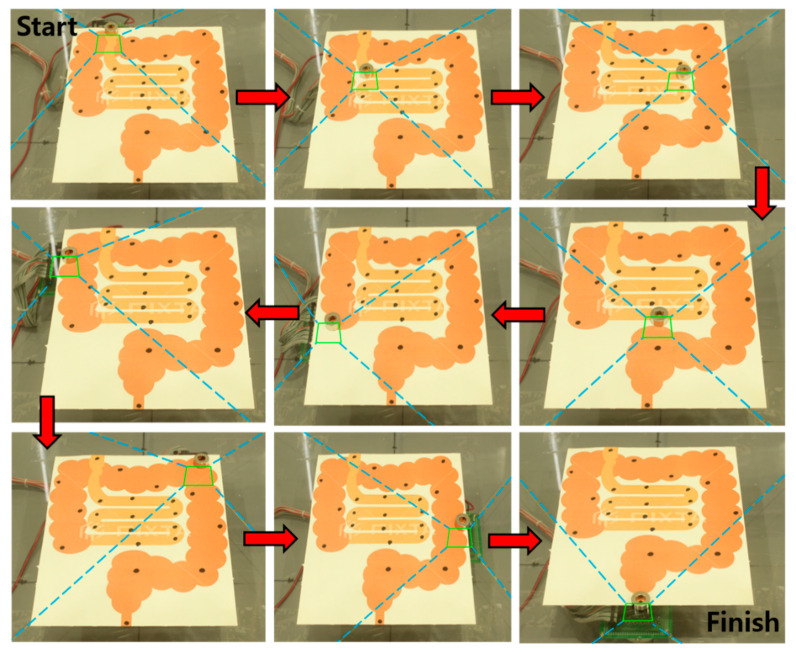
Snapshot of the CDPR tracking system in virtual human small and large intestines.

Figure 13 shows the snapshot of the CDPR tracking the position of the PM while moving the PM sequentially on a path of different point. Blue dotted lines are expressed with EE and cable length because cable changes were not seen well in this figure.

**Figure 14:**
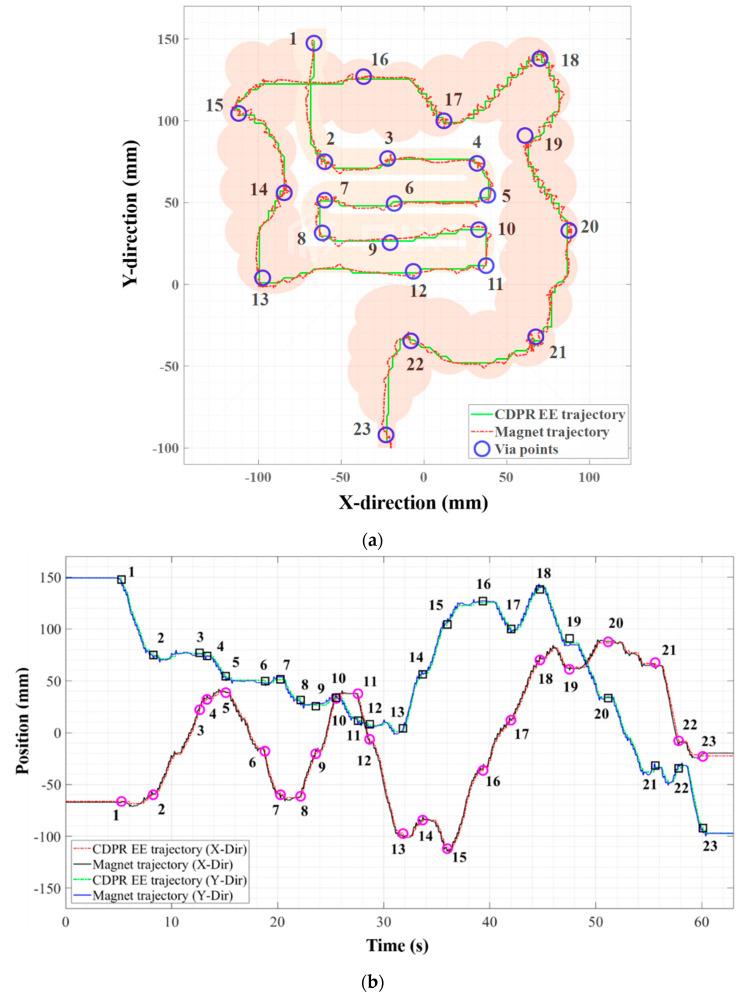
The result of CDPR EE tracking localization performance in 2D space; (**a**) Tracking and localization performance in 3D space and (**b**) Tracking and localization performance in time domain.

Figure 14a shows the result of a posture of the PM controlled to track the illustration of a human intestines model, where the green line is the reference path through forward kinematics of CDPR, and the red line is the PM path through which PM moved, and the blue circle is the random point of the reference path. Figure 14b is a graph of the X, Y and Z position of the PM measured at CDPR within a time span; the values X, Y and Z are the same as the positions in the center of the PM. This result show that localization and tracking performance is adequate even for a persistently moving PM. We moved the PM with average speed of 18.53 mm/s in this experiment. This can verify that the proposed method is sufficient for clinical application by considering the CE’s average speed of 6.13 mm/s [13]. 

#### 3.3.3. Experimental Result

As a result, in experiment at Section 3.3.1, the RMSE values of estimated PM position were 2.922, 1.7208, 1.1625 mm at X, Y, and Z axis, respectively. In the tracking experiment described in Section 3.3.2, the RMSEs for the entire trajectory were 2.238, 2.597 mm at X and Y axis, respectively, where the reference trajectory is marked on the intestinal figure and the real time CDPR EE and magnet position are estimated by the proposed method. The CDPR magnetic position tracking experiment shows that data were received every 10 ms from the TwinCAT 3.1 program, with and error of less than 1.12%. If we consider the peristaltic movement velocity is approximately 2 cm/s in the small intestine, this result shows that the performance of the proposed system is fast and accurate enough to track the location of the CE in a human body.

In addition, the tracking control could be executed within 6 ms in each iteration time that could validate the real time PM localization of the proposed system. In this experiment, we can observe that the error values for X and Y increase significantly as the Z-axis direction increases because the area of the magnetic field decreases and increases the rate of the noise of HES. This is a threshold for measuring the range of HES. Furthermore, this is one of the problems that should be addressed when developing CE tracking systems using CDPR. However, it is possible to develop a CDPR system that allows EE to track the location of the CE in real time. HES is attached to CDPR EE to estimate the location of the CE, and then CDPR will track the estimated position of CE. In this case, it will be possible to determine where the CE is in the human body in real time. However, the results of the position estimation for the Z axis were obtained up to 8 cm using HES only. [44] shows that the average abdominal depth of a typical person is 18 cm. Therefore, the CE tracking system requires a position estimate of at least 20 cm for the Z-axis when the actual CE is inserted into the human body, we will further research how to compensate for the estimated position of the PM on Z-axis by using higher specification HES.

## 4. Discussions and Conclusions

In this study, we have presented a novel robotic localization system based on a planar CDPR and an HES array for the localization of an actively locomotive CE in the human body. By combining the HES array, which is used for tracking the movement of PM in the CE, with the CDPR tracking control, the position of the PM can be obtained by solving the forward kinematics of the CDPR. The size of the current HES array (our HES array size 34 mm × 34 mm) is small compare to the previously developed HES array system ([20] HES device size 70 mm × 70 mm); however, it can cover a wide range of sensing space owing to the CDPR tracking.

The proposed method has a novelty of advanced augmentation of sensor capabilities via robotic system technology with a robotic moving mechanism, which has extensibility and applicability to a wide workspace using a small size sensor. The experimental result shows that the proposed method can extend a sensible volume in space for a particular limited sensing range of a single sensor with small errors. This error might be caused by CDPR control accuracy in each cable at anchor points. Therefore, it is expected that the home position of the EE is inaccurate, resulting in an approximately 1% error in this experiment. Furthermore, the tension control of cables and pulley friction was not considered in this study because CDPR was only used as a tool for estimating the location of magnets. The CDPR works as a robotic motional sensor system that can be extended to a wide space sensor even with the small sensor module.

In the future work, the additional advanced control algorithm for CDPR will be incorporated to improve the accuracy of the proposed system by less than 1%, such as geometric parameter calibration and control mechanism [45,46,47,48]. The location of CDPR was validated in a 1% error by NDI Spectra, and then the accuracy of HES position estimation methodology was evaluated.

Although, a novel concept of PM tracking device system could be validated using planer CDPR and HES, limitations on the height of the current HES were identified. In the future work, we will overcome the limitations of the HES by using sensors that have better magnetic field detection performance of the PM, such as AMR or GMR sensors which have more sensitivity than HES. For the actual position recognition, since the capsule position can be obtained with respect to the X-ray image, the suspicious legion in the long intestine can be identified together while the clinician’s visual inspection. For the 3D reconstruction, because the capsule posture and position are known in terms of the global coordinate, the 3D intestine image can be reconstructed by utilizing the obtained image processing. Finally, we will be developed a tracking device to identify the position of the actual CE through X-ray image and 3D intestine image.

## Figures and Tables

**Table 1:** Results of PM posture recognition by HES; the NDI error is the difference between the NDI measurements and the given trajectory, and the Hall effect sensor error is the difference between the sensor measurement and the given trajectory.

RMSE	NDI				Hall Effect Sensor			
**Helical Trajectory**	X (mm)	Y (mm)		Z (mm)	X (mm)	Y (mm)		Z (mm)
	0.1796	0.1739		0.0441	0.9531	0.8495		1.1382
**Rotation Trajectory**	*θ* (°)		*φ* (°)		*θ* (°)		*φ* (°)	
	0.1232		0.4875		1.1449		1.0408	

**Table 2:** Result of the compared the SNR of the proposed tracking system with the SNR of static condition.

Condition	Signal to Noise Ratio by Vertical Distance (mm)								
	40	45	50	55	60	65	70	75	80
Static (dB)	53.77	39.47	35.36	29.18	27.95	18.19	18.05	16.34	4.55
Moving (dB)	53.77	48.22	41.78	39.07	35.76	35.71	32.76	31.78	29.68

## Nomenclature

$x_{i},y_{i},z_{i}$	Position of the Hall effect sensors
a,b,c	Position of the permanent magnet
m,n,p	Rotational angles of the permanent magnet
$B_{l}$	Magnetic field intensity
$B_{T}$	Constant coefficient
$\mu_{0}$	Air magnetic permeability
$\mu_{r}$	Relative permeability of the medium
$M_{T}$	Magnetization
$T_{l}$	Distance from the vector of permanent magnet to the Hall effect sensors
J	Jacobian matrix
h	Parameter increment
$y_{k}$	Estimated position and rotational value of the permanent magnet
$A_{i}$	Proximal attachment point to the winch
$A_{i}$	Distal attachment point of the end effector
R	Rotation vector
S	Pose of the end effector
